# From SOMAmer-Based Biomarker Discovery to Diagnostic and Clinical Applications: A SOMAmer-Based, Streamlined Multiplex Proteomic Assay

**DOI:** 10.1371/journal.pone.0026332

**Published:** 2011-10-17

**Authors:** Stephan Kraemer, Jonathan D. Vaught, Christopher Bock, Larry Gold, Evaldas Katilius, Tracy R. Keeney, Nancy Kim, Nicholas A. Saccomano, Sheri K. Wilcox, Dom Zichi, Glenn M. Sanders

**Affiliations:** 1 SomaLogic, Inc., Boulder, Colorado, United States of America; 2 Department of Molecular, Cellular and Developmental Biology, University of Colorado Boulder, Boulder, Colorado, United States of America; Deutsches Krebsforschungszentrum, Germany

## Abstract

Recently, we reported a SOMAmer-based, highly multiplexed assay for the purpose of biomarker identification. To enable seamless transition from highly multiplexed biomarker discovery assays to a format suitable and convenient for diagnostic and life-science applications, we developed a streamlined, plate-based version of the assay. The plate-based version of the assay is robust, sensitive (sub-picomolar), rapid, can be highly multiplexed (upwards of 60 analytes), and fully automated. We demonstrate that quantification by microarray-based hybridization, Luminex bead-based methods, and qPCR are each compatible with our platform, further expanding the breadth of proteomic applications for a wide user community.

## Introduction

Anticipation of the utility of analysis of large-scale proteomic content dates back to the realization that phenotypes are manifested by proteins. Successful proteomic analyses, using 2-D gels, were carried out as early as the 1970's [1], and since, interest, technologies, and expectations have continued to develop and escalate. Currently, applications envisioned for proteomic analysis span numerous arenas, including biomarker discovery, life science research, pharmaceutical research, and medical diagnostics. The considerable promise of proteomic measurements in these areas is now being realized, although limitations of current proteomic technologies have impeded development of the more ambitious applications thus far envisioned.

Current proteomic measurement methods tend be limited with respect to throughput, sensitivity, or multiplicity [2]. We previously developed a proteomic platform that promises to surpass current technologies with respect to these limitations. The assay is highly multiplexed, sensitive (sub-picomolar), reproducible, and quantitative [3]. It is based on affinity capture, and therefore has some parallels with antibody-based methods. The assay utilizes synthetic DNA SOMAmers (Slow Off-rate Modified Aptamers) as protein capture reagents rather than antibodies.

SOMAmers are short, single stranded deoxyoligonucleotides. Like aptamers, they are selected *in vitro* from large random libraries for their ability to bind to discrete molecular targets, which can be small molecules, peptides, or proteins [4], [5]. SOMAmers are unlike aptamers in that they bear dU residues that are uniformly functionalized at the 5-position with moieties (e.g. benzyl, 2-napthyl, or 3-indolyl-carboxamide) that can participate in interactions with target molecules as well as form novel secondary and tertiary structural motifs within the SOMAmer itself ([6] data not shown). Nuclease resistance and selection success rates [3] are greatly improved over aptamers, and affinities are comparable to antibodies.

Hence, SOMAmers bear significant promise as synthetic protein-binding reagents [5], [6], [7]. In particular, we find that problems of capture reagent cross-reactivity and non-specific adsorption to surfaces are diminished or absent in our SOMAmer-based protein measurement assays. These are issues that limit the intrinsic multiplexing capability of antibody-based proteomic assays to 30–50 analytes [2]. In contrast, at the time of this writing, our SOMAmer-based, high-content biomarker discovery platform reliably measures more than one thousand protein analytes in a single sample ([3] data not shown). As yet, we do not anticipate an upper limit to the multiplex capacity of this platform.

Here we present a streamlined, microtiter plate-based version of our multiplex SOMAmer-based proteomics discovery assay. The assay is intended to provide a rapid, efficient and seamless transition from SOMAmer-based protein biomarker panel identification [3], [10], [11] to routine proteomic measurements. Potential applications include *in vitro* diagnostic assays, assays intended to facilitate clinical drug development, and indeed, any proteomic measurement that might otherwise be carried out by ELISA.

**Figure 1:**
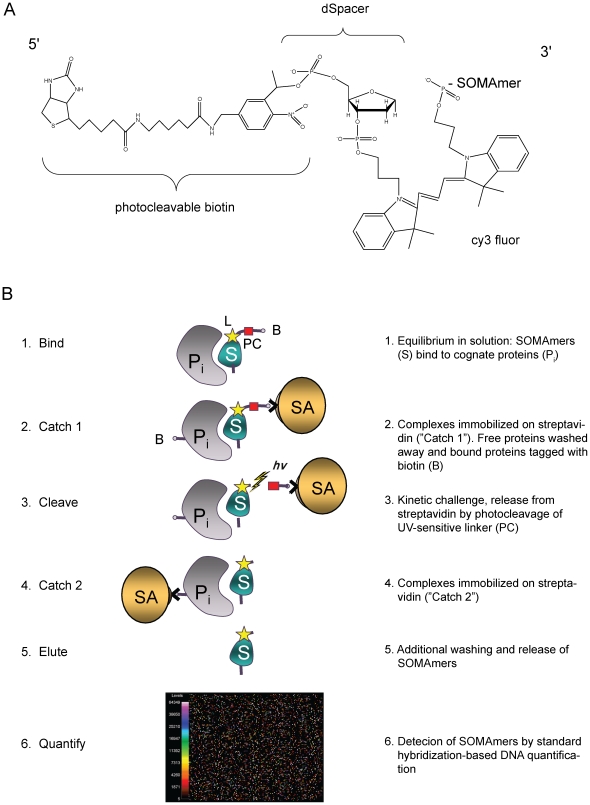
SOMAmer-based assay reagent and assay principles. The primary analyte capture reagent and quantified component consists of an analyte-specific SOMAmer coupled to a Cy3 moiety and a biotin group joined to the fluorophore-substituted SOMAmer through a photocleavable linker (Panel A). The principal features of the assay consist of equilibration of capture reagent and analyte mixture in solution, followed by immobilization of the entire capture reagent population on immobilized streptavidin through the biotin moieties of the capture reagent population. The immobilized capture reagent population, including analyte/capture reagent complexes, is washed to remove non-complexed proteins. Immobilized protein analytes are then biotinylated using a conventional amine-reactive biotinylation reagent. The entire capture reagent population, including biotinylated analyte/capture reagent complexes, is then released into solution via photocleavage. Biotinylated analyte/capture reagent complexes are exclusively captured on immobilized streptavidin via the biotin moieties appended to the analyte population. Washes remove the capture reagent population at large, leaving only analyte/capture reagent complexes. The remaining capture reagent population is a surrogate for the analyte/capture reagent population. This material is eluted from immobilized analytes and quantified via conventional DNA quantification methods.

At the core of our SOMAmer-based protein measurement assays is an analyte capture reagent that consists of a fully synthetic SOMAmer coupled to a biotin moiety through a photocleavable linker (referred to as a “PB-SOMAmer”, Figure 1A). The biotin moiety permits binding to the streptavidin supports used for immobilization and wash steps, while the photocleavable linker permits release of the SOMAmer into solution after washing. A Cy3 fluorophore built into the capture reagents used in this study permits quantification by means of commercially available slide-based microarray hybridization systems, but is not required for all formats of the assay.

The assays themselves consist of a binding step in which PB-SOMAmers and analytes are equilibrated in solution (Figure 1B Panel 1), followed by immobilization of all PB-SOMAmers on a streptavidin-substituted support (Figure 1B Panel 2, “Catch-1”). Subsequent washes remove proteins that are not stably complexed with PB-SOMAmers. Proteins immobilized through interaction with bound PB-SOMAmers are biotinylated with an amine-reactive biotinylation reagent (N-hydroxysuccinimide-PEO_4_-biotin). After further washes, the entire SOMAmer population, including analyte-SOMAmer complexes, is released into solution via long-wave ultraviolet light-catalyzed cleavage of the biotin-bearing photocleavable linker (Figure 1B Panel 3). The biotinylated analyte-SOMAmer complexes are then selectively captured on another streptavidin support (Figure 1B Panel 4, “Catch-2”) and the remaining, non-complexed SOMAmers are washed away. Finally, analyte-bound SOMAmers are eluted by disrupting the affinity interaction (Figure 1B Panel 5). Eluted SOMAmers are surrogates for analyte concentrations that can be quantified by standard DNA-quantification methods, for example, qPCR, or hybridization to microarrays (Figure 1B Panel 6). Conversion of arbitrary DNA measurement units that reflect protein concentrations into actual protein concentration units is accomplished through use of standard curves.

## Results

To develop the plate-based SOMAPanel assay, a model multiplex consisting of nine PB-SOMAmers specific for the proteins IL-8, tPA, resistin, MIP-4, MMP-7, MMP-9, RANTES, MCP-1, and Lipocalin 2, and twenty control PB-SOMAmers, was assembled. These target analytes were chosen arbitrarily from our complete SOMAmer menu as representative of three broad ranges of abundance in plasma or serum, and because their PB-SOMAmer capture reagents had been rigorously demonstrated as specific for their respective analytes by pull-down assay (Supporting Figure S1). The twenty control PB-SOMAmers consist of a mixture of sequences designed to have particular secondary structure motifs found in bona fide SOMAmers (for example, stem-loop or G-quartet) and sequences selected to recognize non-human proteins (for example, Green Fluorescent Protein (GFP) from *Aequorea victoria*, ά-hemolysin from *Staphylococcus aureus*, firefly luciferase, keyhole limpet cyanin, and glutaredoxin from *Escherichia coli*. These were used to monitor non-specific plasma-dependent SOMAmer signaling in the course of assay development (data not shown). This SOMAmer panel will be referred to as a “nine-plex” although it consists of nine capture reagents proper plus additional controls.

### Features of the plate-based SOMAPanel assay

**Figure 2:**
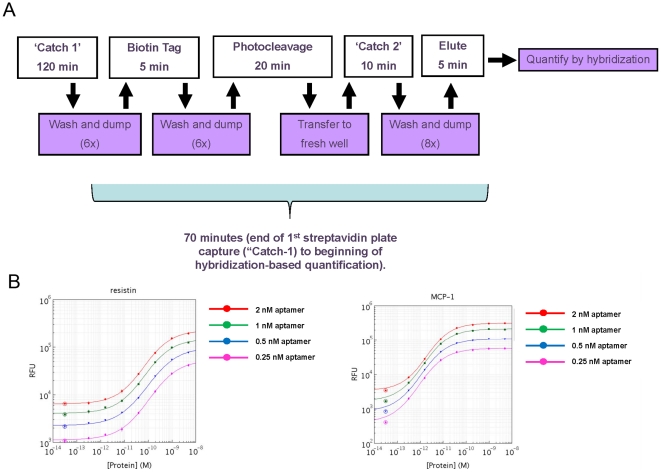
Diagram of manual plate-based assay steps and dose-response curves generated in manual plate-based assay format. The five steps of the manual plate-based assay - equilibration, biotinylation, photocleavage, and elution, are punctuated by three wash-and-dump cycles and one liquid transfer. The total processing time is about 70 minutes (Panel A). A set of dose response curve generated in a nine-plex manual assay format at various capture reagent concentrations (Panel B). Dose-response curves were generated by spiking analytes into plasma at the indicated concentrations. Shown are resistin and MCP-1. Dose-response curves of MIP-4, RANTES, MMP-9, MMP-7, Lipocalin 2, tPA, and IL-8 can be found in Supporting Materials.

Substitution of streptavidin plates for streptavidin-agarose beads and magnetic streptavidin beads eliminates both vacuum filtration and magnetic separation from the assay protocol. In manual form, the assay becomes a “wash and dump” procedure that is reminiscent of an ELISA-based assay. The hands-on processing time, which excludes equilibration and the initial streptavidin capture (3.5 hours), is roughly 70 minutes. A diagram of the assay steps is shown in Figure 2A. It should be noted that equilibration time is a flexible parameter dictated by analyte abundance and requirements for sensitivity. We have chosen a long equilibration time here to maximize sensitivity for extremely sparse analytes. Very acceptable results have been obtained with equilibration times as short as 15 minutes (data not shown).

Dose-response curves with purified analytes generated in the plate-based assay performed manually are shown in Figure 2B. This 9-plex measurement compared assay response to increasing spiked-in analyte concentration as a function of PB-SOMAmer concentration in the presence of plasma. The dynamic range of the assay is, for analytes shown, roughly 3 logs. In general, little sensitivity or dynamic range is gained by elevated SOMAmer concentrations. We have chosen an intermediate concentration, 0.5 nM for each PB-SOMAmer, for the work shown here, though it is apparent from the curves that more or less PB-SOMAmer can be used in this particular analyte panel without significant penalty.

### Semi-automation of the plate-based assay

It was anticipated that the “wash and dump” nature of the plate-based assay would permit automation using commercially available, relatively low-cost instrumentation intended for ELISA. The nature of the assay suggested that the additional capability of multiple reagent addition would permit near-complete automation. The commercially available BioTek EL406 was selected for this capacity. In addition to its conventional plate-washing capability, the EL406 supports addition of up to six different reagents, which is the number of solutions used in this assay.

We adapted the manual protocol for use with the EL406. The resulting semi-automated, plate-based assay protocol proved considerably more rapid and convenient than the semi-automated bead-based assay. The post-equilibration processing time was reduced from one hundred fifty minutes to fifty minutes. Hands-on operations for the semi-automated assay became limited to movement of plates from plate washer to UV lamp and back and transfer of samples from one plate to another after photocleavage.

To assess assay performance, we generated precision profiles for each analyte in this assay format, and compared these with precision profiles generated in our biomarker discovery assay format. Precision profiles provide a quantitative measure of assay performance. In a precision profile, the coefficient of variation (%CV, determined from 8 replicates of each data point in this case) is plotted as a function of analyte concentration. Upper and lower limits of quantification (ULOQ and LLOQ, respectively), defined in this case as analyte concentrations at which %CV's exceed 20% may be established from these plots. Quantification range is defined as the range of analyte concentrations for which the %CV is less than 20%.

**Figure 3:**
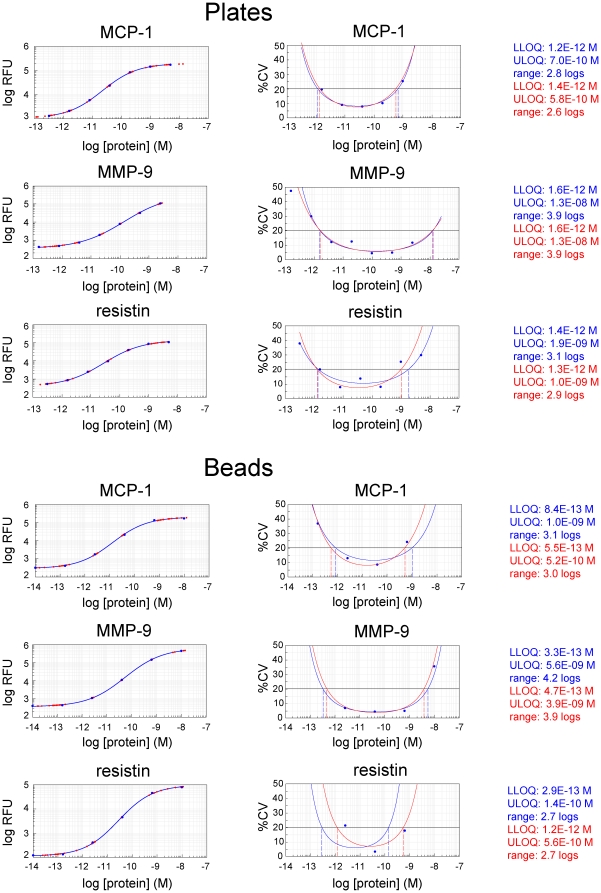
Precision profiles and limits of quantification of plate- and bead-based assays. Eight individual measurements of fluorescent signal as a function of analyte concentration in buffer were made for each of nine analytes in multiplexed format. For the dose-response curves (left of each panel), the average RFU at each concentration is denoted by the blue markers and the eight individual measurements used to compute each average are denoted by the red markers plotted on the four parameter curve fit (solid blue line). Precision profiles (right of each panel) were computed with two different methods: (1) by calculating the variance in computed concentrations (blue, bottom left of each panel) and (2) by calculating the variance in log RFU (assay response, top right of each panel) combined with the slope of the standard curve (red). Limits of quantification were defined as points on the curves where the coefficient of variation (CV) exceeded 20%. Panels A, C, and E were generated in plate-based format. Panels B, D, and F were generated in bead-based format. Analytes measured were MCP-1 (Panels A and B), MMP9 (Panel C and D), resistin (Panels E and F) tPA (Figure S1B), MMP-7 (Figure S2B), IL-8 (Figure S2A), Lipocalin 2 (Figure S2A), MIP-4 (Figure S2A), Protein S (Figure S2B) and RANTES (Figure S2B).

Comparison of ULOQ's, LLOQ's, and quantification ranges measured in the 9-plex, semi-automated, plate-based assay format with those measured in the bead-based biomarker discovery assay format revealed little loss in sensitivity, stability, or dynamic range. (Figure 3 and Supporting Figure S2, compare left and right panels). We conclude that assay performance of the two assay formats is similar.

A summary comparison of the properties of the two assays is presented in Table 1.

**Table 1:** Comparison of automated bead-based Discovery-plex and plate-based SOMAPanel assay formats.

Metric	Bead-based Discovery Assay	Plate-based SOMAPanel Targeted Assay
Partitioning Method (Capacity)	Catch 1 – SA Agarose Beads (>1000-plex)	Catch 1 & 2 – SA plates (∼200-plex)
Up-front Prep (Time)	Bead prep (∼30 minutes) Robotic setup	None
Post-equilibration processing time	∼150 minutes	∼50 minutes
Throughput	96 samples/day/FTE	384 samples/day/FTE
Manual operation	Yes	Yes
Average LLOQ	<1 pM	<2 pM
Coefficient of variation	∼5%	∼7%
Automation instrumentation	Biomek FX with modifications	Stock BioTek EL406 plate washer

### Nucleic acid quantification schemes

The bead-based biomarker discovery assay and plate-based experiments shown up to this point use a nucleic acid quantification system based on hybridization to printed microarrays from Agilent to quantify SOMAmers in the final assay eluate. This system currently has the capacity to quantitatively measure more than 3000 analytes per sample, and even higher levels of multiplexing are anticipated. It has proven sensitive and convenient for very highly multiplexed biomarker discovery applications. However, many labs have invested in other potentially suitable hybridization-based nucleic acid quantification instrumentation. Hence, we compared an alternative bead-based nucleic acid quantification platform with Agilent microarrays with respect to compatibility with our SOMAmer-based multiplex assay.

We generated precision profiles and determined limits of quantification of 9 analytes in multiplex format, in exactly the same manner as in Figure 3. We split the final eluates into two parts and independently determined limits of quantification using Agilent microarrays and the Luminex bead-based system as a final readout (Table 2). We found that sensitivity and dynamic ranges are roughly comparable between the two platforms, although Luminex was slightly less sensitive than Agilent (Table 2, compare columns 2 and 3), and exhibited slightly elevated upper limits of quantification (Table 2, compare columns 4 and 5).

**Table 2:** Comparison of slide-based and bead-based read-out formats.

	Lower Limit of Quantification (pM)		Upper Limit of Quantification (pM)		Quantification Range (logs)	
Analyte	Agilent	Luminex	Agilent	Luminex	Agilent	Luminex
IL-8	0.32	0.5	210	240	2.8	2.7
MIP-4	0.66	2.0	3,600	1000,000	3.8	5.7
Lipocalin-2	0.83	0.78	260	1,500	2.5	3.3
MCP-1	1.2	1.8	700	1,500	2.8	3.9
RANTES	1.8	3.5	420	360	2.4	2.0
MMP-7	1.9	6.8	550	1,100	2.5	2.2
resistin	1.4	1.8	1,900	4,400	3.1	3.4
MMP-9	1.6	5.4	13,000	19,000	3.9	3.5
tPA	1.2	3.2	1,300	1,400	3.1	2.7

Our biomarker discovery efforts have revealed that analyte concentration differences that distinguish case from control populations are often subtle. Indeed, we have discovered useful biomarkers that differ by as little as twenty percent between case and control [9]. To determine whether the plate-based assay in combination with a Luminex bead-based nucleic acid readout is suitable for measurements involving such subtle analyte concentration differences in the region of endogenous levels, we spiked in analytes in 20% increments, in quintuplicate, in the regions of analyte signal previously measured in serum titrations (Supporting Figure S4). It should be noted that listed analyte concentrations are nominal, based on protein mass as noted by the manufacturer without reference to purity, and hence cannot be used as standards to infer actual endogenous concentrations.

**Figure 4:**
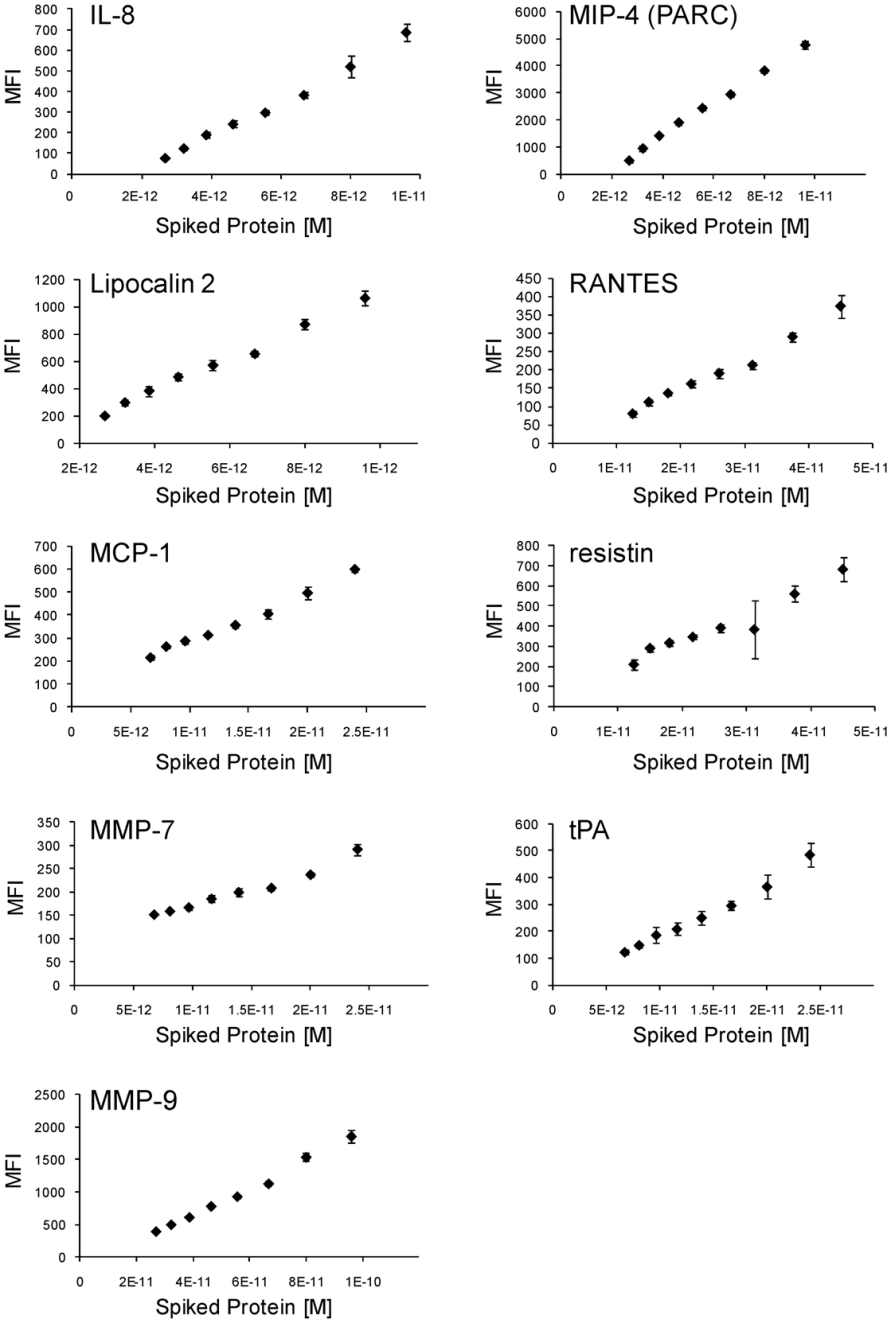
High-resolution titration of analytes. Analytes were titrated in 20% concentration increments in the region of signal generated by serum without spikes. Assay eluates were quantified by Luminex bead hybridization.

We find that even at these low levels of signal, subtle changes in concentration can be measured with good precision (Figure 4). The average CV for all analytes was 6.1%, with linear responses in the ranges tested.

### Validating the plate-based front end with alternative back-end readouts

We wished to determine whether the plate-based assay, in combination with a bead-based nucleic acid (SOMAmer) readout, is sufficiently sensitive and robust to separate case and control populations within a clinical sample set. This is a commercial diagnostic application anticipated for SOMAPanel assays. To this end we performed an experiment in which various analytes were spiked at levels comparable to those we have encountered in the course of biomarker discovery into a collection of individual serum samples, effectively creating a mock disease signature in a population of samples. The ability to distinguish differential expression of analytes, both up and down with respect to the control population, forms a practical criterion for the adequacy of the assay to discern target responses against the backdrop of individual sample variance.

Serum samples from twenty-four healthy controls were used to create a protein signature with both “up-” and “down-regulated” analytes. Two aliquots of each sample were used to produce a separate control and a case population by adding analytes to each group. Spikes into the twenty-four samples comprising the control group will result in “down-regulated” measurements in the case population while spikes into the case population will result in “up-regulation”. Spike concentrations were chosen to produce analyte signals that differed by 1–2 standard deviations from the means of the population distributions as determined from the twenty-four individuals. These modest concentration differences were intended to mimic real-world case and control samples, rather than simply achieve clean separation of two populations. We spiked three analytes into the control samples (tPA, MMP-9, and Lipocalin 2), and four analytes in the case samples (IL-8, MCP-1, resistin and RANTES). The model multiplex assay was used to explore differential expression in this set of mock case and control samples.

**Figure 5:**
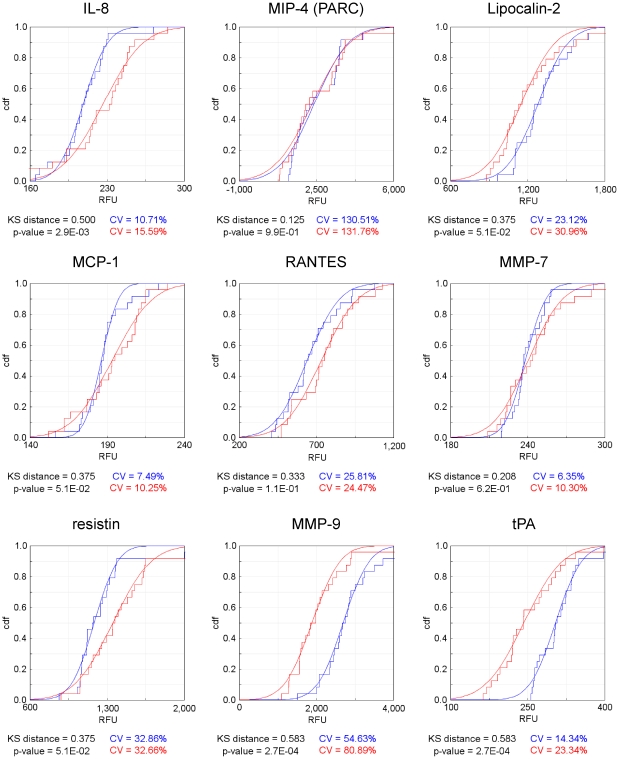
Differential expression between case and control populations. Twenty-four case samples and twenty-four control samples were measured using the model 9-plex plate-based assay. Empirical CDFs were constructed for the control (blue) and case (red) populations separately for each analyte and are displayed in panels a–i. Spikes into the control samples (tPA, MMP-9, and Lipocalin 2) result in clear “down-regulation”, spikes into case samples (IL-8, MCP-1, resistin and RANTES) result in clear “up-regulation” and the two analytes not spiked (MIP-4 and MMP-7) show no differential expression.

The results are presented in Figure 5. Cumulative distribution functions (CDFs) were constructed separately for the case and control populations for each of the nine analytes. The three analytes spiked into the control group result in clearly identified “down-regulation”, while the four analytes spiked into the case group appear as “up-regulation” in our mock protein signature. The two analytes for which no spikes were added, MIP-4, and MMP-7, display no differential expression, attesting to the specificity of the SOMAmer assay. The magnitudes of the spiked proteins were relatively small to result in mostly overlapping distributions between case and control populations yet with discernable differences that are comparable to those observed in actual case/control proteomic studies [10]. The plate-based SOMAmer assay performed well in this model multiplex diagnostic application.

Finally, to verify that our results with spiked proteins can be recapitulated with native analytes, we performed a reproducibility study with the plate-based assay, using a Luminex read-out and unspiked plasma samples. Eight identical pooled plasma samples and plasma samples from 24 individuals were measured in 3 independent runs.

Intra- and inter-run coefficients of variation (CV's) within the 8 pooled plasma samples were calculated. The median intra-run CV was 11.4%, while the median inter-run CV was 11.9% (Table 3). These values are well within our hypothetical case/control variation of 20%.

**Table 3:** Reproducibility study using Luminex readout.

Analyte	Mean RFU (std dev) (8 calibrators, 3 runs)	Inter-run CV (%) (8 calibrators, 3 runs)	Intra-run CV (%) (8 calibrators)	Median Spearman's Rho (24 individuals)	P-value
IL-8	104 (12)	11.6	11.8	0.471	2.0×10^−2^
MIP-4	808 (87)	10.9	9.6	0.875	2.3×10^−8^
Lipocalin-2	609 (105)	17.3	12.4	0.495	1.4×10^−2^
MCP-1	203 (16.3)	8.1	8.2	0.617	1.3×10^−3^
RANTES	409 (47)	11.4	10.3	0.795	3.5×10^−6^
MMP-7	193 (19.8)	10.3	11.9	0.590	2.4×10^−3^
resistin	153 (20)	11.6	11.8	0.449	2.8×10^−2^
MMP-9	168 (17.2)	10.3	8.4	0.829	5.7×10^−7^

Spearman's rank correlation coefficients (Spearman's Rho's) were calculated for each analyte within the 24 individuals between runs 1 and 2, 1 and 3, and 2 and 3 (Table 3). Spearman's Rho's provide a metric for reproducibility of subtle individual variations, and ultimately, a surrogate measurement for ability to distinguish case from control in test populations. It should be noted that in the case of close population distributions (those within assay variation), Spearman's correlations will break down, and thus Spearman's correlations within a normal population can represent an artificially high bar for ability to distinguish test and control. Conversely, wide population distributions can generate very high Spearman's correlations. Here, we present Spearman's correlations as a simple demonstration that our assay can reproducibly distinguish subtle variations in analyte signal in native, unspiked plasma samples.

## Discussion

We developed a streamlined multiplexed SOMAmer-based assay that is robust, sensitive, and quantitative. It is designed to enable the translation of discovery biomarker panels into robust diagnostic products and to facilitate use of small panels during clinical development of drugs. The ease of use of the assay is roughly comparable to that of single-analyte ELISA. The assay is easily and inexpensively automated. Throughput can be made relatively high and sample volumes are quite small (∼15 µL). Equipment and materials for the assay are commercially available from several sources. The final readout can be made inexpensive and scaled according to analyte number by use of a commercially available, bead-based nucleic acid quantification system.

Recently, we have identified several biomarker panels with potential diagnostic applications for chronic kidney disease [3], lung cancer [10], mesothelioma, and pancreatic cancer [in preparation]. Use of these biomarker panels in diagnostic applications will require the measurement of perhaps 9–15 analytes in a single sample. The streamlined assay presented here permits seamless transition from such biomarker panels identified in SOMAScan-based studies to actual diagnostic applications. We have demonstrated such utility by spiking analytes into a sample population to produce typical differential expression observed in proteomic studies of case/control groups. We have further demonstrated that even the subtle variations in native analyte levels within a normal population can be reproducibly distinguished.

We have found that the bead-based nucleic acid quantification system from Luminex can be used for final readout without significant performance penalty. This is advantageous in that it permits scaling of the final readout to the number of analytes to be measured. As such, the assay can be made more economical for applications involving specific small analyte panels. Moreover, the demonstration that several readout platforms may be used will render the SOMAmer-based assay easily accessible to groups and institutions that already possess other gene expression measurement platforms.

We have also explored the use of real-time quantitative PCR (qPCR) as a back-end read-out for the plate- and bead-based assays. As might be expected, qPCR is exquisitely sensitive, and can be made reproducible and quantitative with appropriate optimization (Figure S3 and data not shown). We conclude that qPCR is a viable back-end readout option for the SOMAmer-based assay presented here, as is any other nucleic acid quantification scheme. It is certainly suitable for experimentation as well as routine assays in labs that possess the necessary equipment.

We have only briefly explored the upper limits of multiplex capacity of the plate-based assay but have verified that at least 60 analytes, selected from a lung cancer panel identified as biomarkers in an 836-plex SOMAScan assay, may be multiplexed without optimization (Figure S5).

The use of SOMAmers as capture reagents carries advantages over traditional antibody-based immunoassays. The synthetic nature of SOMAmers ensures uniformity and availability. Customization of the affinity reagent is routine, relying only on the availability of the appropriate phosphoramidites. SOMAmers are as chemically stable as DNA and resistant to at least 10 freeze-thaw cycles when buffered (Steve Wolk, pers. comm.). Heat denaturation of SOMAmers is completely reversible and in fact SOMAmers are routinely heated to 95 C prior to use (see Materials and Methods). Custom generation of SOMAmers to protein targets is generally rapid and inexpensive compared to antibodies. The intrinsic limitations of multiplex capabilities of antibodies are greatly diminished with SOMAmers. To date, we have successfully multiplexed up to 1034 SOMAmer measurements in a single 15 µL sample and do not anticipate an upper limit on multiplexing.

## Materials and Methods

### Purchased reagents

HEPES, NaCl, KCl, EDTA, EGTA, MgCl_2_ and Tween-20 were purchased from Fisher Biosciences. Dextran sulfate sodium salt (DxSO_4_), nominally 8000 molecular weight, was purchased from AIC and dialyzed against deionized water for at least 20 hours with one exchange. KOD EX DNA polymerase was purchased from VWR. Tetramethylammonium chloride and CAPSO were purchased from Sigma-Aldrich and streptavidin-phycoerythrin (SAPE) were purchased from Moss Inc. 4-(2-Aminoethyl)-benzenesulfonylfluoride hydrochloride (AEBSF) was purchased from Gold Biotechnology. Streptavidin-coated 96-well plates were purchased from Thermo Scientific (Pierce Streptavidin Coated Plates HBC, clear, 96-well, product number 15500 or 15501). NHS-PEO4-biotin was purchased from Thermo Scientific (EZ-Link NHS-PEO4-Biotin, product number 21329), dissolved in anhydrous DMSO, and stored frozen in single-use aliquots. IL-8, MIP-4, Lipocalin-2, RANTES, MMP-7, and MMP-9 were purchased from R&D Systems. Resistin and MCP-1 were purchased from PeproTech, and tPA was purchased from VWR.

### Nucleic acids

Conventional (including amine- and biotin-substituted) oligodeoxynucleotides were purchased from Integrated DNA Technologies (IDT). Z-Block is a single-stranded oligodeoxynucleotide of sequence 5′- (AC-BnBn)_7_-AC-3′, where Bn indicates a benzyl-substituted deoxyuridine residue. Z-block was synthesized in-house, using conventional phosphoramidite chemistry. SOMAmer capture reagents were synthesized in-house by conventional phosphoramidite chemistry, and purified on a 21.5×75 mm PRP-3 column, operating at 80°C on a Waters Autopurification 2767 system (or Waters 600 series semi-automated system), using a timberline TL-600 or TL-150 heater and a gradient of triethylammonium bicarbonate (TEAB) / ACN to elute product. Detection was performed at 260 nm and fractions were collected across the main peak prior to pooling best fractions.

### Buffers

Buffer SB18 is composed of 40 mM HEPES, 101 mM NaCl, 5 mM KCl, 5 mM MgCl_2_, and 0.05% (v/v) Tween 20 adjusted to pH 7.5 with NaOH. Buffer SB17 is SB18 supplemented with 1 mM trisodium EDTA. Buffer PB1 is composed of 10 mM HEPES, 101 mM NaCl, 5 mM KCl, 5 mM MgCl_2_, 1 mM trisodium EDTA and 0.05% (v/v) Tween-20 adjusted to pH 7.5 with NaOH. CAPSO elution buffer consists of 100 mM CAPSO pH 10.0 and 1 M NaCl. Neutralization buffer consists of 500 mM HEPES, 500 mM HCl, and 0.05% (v/v) Tween-20. Agilent Hybridization Buffer is a proprietary formulation that is supplied as part of a kit (Oligo aCGH/ChIP-on-chip Hybridization Kit). Agilent Wash Buffer 1 is a proprietary formulation (Oligo aCGH/ChIP-on-chip Wash Buffer 1, Agilent). Agilent Wash Buffer 2 is a proprietary formulation (Oligo aCGH/ChIP-on-chip Wash Buffer 2, Agilent). TMAC hybridization solution consists of 4.5 M tetramethylammonium chloride, 6 mM trisodium EDTA, 75 mM Tris-HCl (pH 8.0), and 0.15% (v/v) Sarkosyl. KOD buffer (10-fold concentrated) consists of 1200 mM Tris-HCl, 15 mM MgSO_4_, 100 mM KCl, 60 mM (NH_4_)_2_SO_4_, 1% v/v Triton-X 100 and 1 mg/mL BSA.

### Sample preparation

Serum (stored at −80°C in 100 µL aliquots), was thawed in a 25°C water bath for 10 minutes, then stored on ice prior to sample dilution. Samples were mixed by gentle vortexing for 8 seconds. A 6% serum sample solution was prepared by dilution into 0.94× SB17 supplemented with 0.6 mM MgCl_2_, 1 mM trisodium EGTA, 0.8 mM AEBSF, and 2 µM Z-Block. A portion of the 6% serum stock solution was diluted 10-fold in SB17 to create a 0.6% serum stock. 6% and 0.6% stocks are used to detect high- and low-abundance analytes, respectively.

### Capture reagent (SOMAmer) and streptavidin plate preparation

SOMAmers were grouped into 2 mixes according to the relative abundance of their cognate analytes. SOMAmers consigned to the low-abundance group were those that recognize IL-8, MMP-7, resistin, and tPA. SOMAmers consigned to the high-abundance group were those that recognize Lipocalin-2, MCP-1, MIP-4 (PARC), MMP-9, and RANTES. Stock concentrations were 4 nM in each SOMAmer, and the final concentration of each SOMAmer was 0.5 nM. SOMAmer stock mixes were diluted 4-fold in SB17 buffer, heated to 95°C for 5 min and cooled to 37°C over a 15 minute period prior to use. This denaturation-renaturation cycle is intended to normalize SOMAmer conformer distributions and thus ensure reproducible SOMAmer activity in spite of variable histories. Streptavidin plates were washed twice with 150 µL buffer PB1 prior to use.

### Equilibration and plate capture

Heat-cooled 2× SOMAmer mixes (55 µL) were combined with an equal volume of 6% or 0.6% serum dilutions, producing equilibration mixes containing 3% and 0.3% serum. The plates were sealed with a Silicone Sealing Mat (Axymat Silicone sealing mat, VWR) and incubated for 1.5 h at 37°C. Equilibration mixes were then transferred to the wells of a washed 96-well streptavidin plate and further incubated on an Eppendorf Thermomixer set at 37°C, with shaking at 800 rpm, for two hours.

### Manual Assay

Unless otherwise specified, liquid was removed by dumping, followed by two taps onto layered paper towels. Wash volumes were 150 µL and all shaking incubations were done on an Eppendorf Thermomixer set at 25°C, 800 rpm. Equilibration mixes were removed by pipetting, and plates washed twice for 1 minute with buffer PB1 supplemented with 1 mM dextran sulfate and 500 µM biotin, then 4 times for 15 seconds with buffer PB1. A freshly made solution of 1 mM NHS-PEO_4_-biotin in buffer PB1 (150 µL/well) was added, and plates incubated for 5 minutes with shaking. The NHS-biotin solution was removed, and plates washed 3 times with buffer PB1 supplemented with 20 mM glycine, and 3 times with buffer PB1. Eighty-five µL of buffer PB1 supplemented with 1 mM DxSO_4_ were then added to each well, and plates were irradiated under a BlackRay UV lamp (nominal wavelength 365 nm) at a distance of 5 cm for 20 minutes with shaking. Samples were transferred to a fresh, washed streptavidin-coated plate, or an unused well of the existing washed streptavidin plate, combining high and low sample dilution mixtures into a single well. Samples were incubated at room temperature with shaking for 10 minutes. Unadsorbed material was removed and the plates washed 8 times for 15 seconds each with buffer PB1 supplemented with 30% glycerol. Plates were then washed once with buffer PB1. SOMAmers were eluted for 5 minutes at room temperature with 100 µL CAPSO elution buffer. 90 µL of the eluate was transferred to a 96-well HybAid plate and 10 µL neutralization buffer was added.

### Semi-Automated Assay

Streptavidin plates bearing adsorbed equilibration mixes were placed on the deck of a BioTek EL406 plate washer, which had been programmed to perform the following steps: unadsorbed material is removed by aspiration, and wells are washed 4 times with 300 µL of buffer PB1 supplemented with 1 mM dextran sulfate and 500 µM biotin. Wells are then washed 3 times with 300 µL buffer PB1. One hundred fifty µL of a freshly prepared (from a 100 mM stock in DMSO) solution of 1 mM NHS-PEO_4_-biotin in buffer PB1 is added. Plates are incubated for 5 minutes with shaking. Liquid is aspirated, and wells were washed 8 times with 300 µL buffer PB1 supplemented with 10 mM glycine. One hundred µL of buffer PB1 supplemented with 1 mM dextran sulfate are added. After these automated steps, plates were removed from the plate washer and placed on a thermoshaker mounted under a UV light source (BlackRay, nominal wavelength 365 nm) at a distance of 5 cm for 20 minutes. The thermoshaker was set at 800 rpm and 25°C. After 20 minutes irradiation, samples were manually transferred to a fresh, washed streptavidin plate (or to an unused well of the existing washed plate). High-abundance (3% serum+3% SOMAmer mix) and low-abundance reaction mixes (0.3% serum+0.3% SOMAmer mix) were combined into a single well at this point. This “Catch-2” plate was placed on the deck of BioTek EL406 plate washer, which had been programmed to perform the following steps: the plate was incubated for 10 minutes with shaking. Liquid is aspirated, and wells are washed 21 times with 300 µL buffer PB1 supplemented with 30% glycerol. Wells are washed 5 times with 300 µL buffer PB1, and the final wash is aspirated. One hundred µL CAPSO elution buffer are added, and SOMAmers are eluted for 5 minutes with shaking. Following these automated steps, the plate was then removed from the deck of the plate washer, and 90 µL aliquots of the samples were transferred manually to the wells of a HybAid 96-well plate that contained 10 µL neutralization buffer.

### Hybridization to custom Agilent 8×15k microarrays

24 µL of the neutralized eluate were transferred to a new 96-well plate and 6 µL of 10× Agilent Block (Oligo aCGH/ChIP-on-chip Hybridization Kit, Large Volume, Agilent 5188–5380), containing a set of hybridization controls composed of 10 Cy3 SOMAmers was added to each well. Thirty µL 2× Agilent Hybridization buffer were added to each sample and mixed. Forty µL of the resulting hybridization solution were manually pipetted into each “well” of the hybridization gasket slide (Hybridization Gasket Slide, 8-microarray per slide format, Agilent). Custom Agilent microarray slides, bearing 10 probes per array complementary to 40 nucleotide random region of each SOMAmer with a 20× dT linker, were placed onto the gasket slides according to the manufacturers' protocol. The assembly (Hybridization Chamber Kit – SureHyb-enabled, Agilent) was clamped and incubated for 19 hours at 60°C while rotating at 20 rpm.

### Post Hybridization Washing

Approximately 400 mL Agilent Wash Buffer 1 was placed into each of two separate glass staining dishes. Slides (no more than two at a time) were disassembled and separated while submerged in Wash Buffer 1, then transferred to a slide rack in a second staining dish also containing Wash Buffer 1. Slides were incubated for an additional 5 minutes in Wash Buffer 1 with stirring. Slides were transferred to Wash Buffer 2 pre-equilibrated to 37°C and incubated for 5 minutes with stirring. Slides were transferred to a fourth staining dish containing acetonitrile, and incubated for 5 minutes with stirring.

### Microarray Imaging

Microarray slides were imaged with an Agilent G2565CA Microarray Scanner System, using the Cy3-channel at 5 µm resolution at 100% PMT setting, and the XRD option enabled at 0.05. The resulting TIFF images were processed using Agilent feature extraction software version 10.5.1.1 with the GE1_105_Dec08 protocol. Primary Agilent data is available as Supplementary Information (Figure S6).

### Luminex probe design

Probes immobilized to beads bore 40 deoxynucleotides complementary to the 3′ end of the 40 nucleotide random region of the target SOMAmer. The SOMAmer complementary region was coupled to Luminex Microspheres through a hexaethyleneglycol (HEG) linker bearing a 5′ amino terminus. Biotinylated detection deoxyoligonucleotides consisted of 17–21 deoxynucleotides complementary to the 5′ primer region of target SOMAmers. Biotin moieties were appended to the 3′ ends of detection oligos.

### Coupling of probes to Luminex Microspheres

Probes were coupled to Luminex Microplex Microspheres essentially per the manufacturer's instructions, but with the following modifications: amino-terminal oligonucleotide amounts were 0.08 nMol per 2.5×10^6^ microspheres, and the second EDC addition was 5 µL at 10 mg/mL. Coupling reactions were performed in an Eppendorf ThermoShaker set at 25°C and 600 rpm.

### Microsphere hybridization

Microsphere stock solutions (about 40000 microspheres/µL) were vortexed and sonicated in a Health Sonics ultrasonic cleaner (Model: T1.9C) for 60 seconds to suspend the microspheres. Suspended microspheres were diluted to 2000 microspheres per reaction in 1.5× TMAC hybridization solutions and mixed by vortexing and sonication. Thirty-three µL per reaction of the bead mixture were transferred into a 96-well HybAid plate. Seven µL of 15 nM biotinylated detection oligonucleotide stock in 1× TE buffer were added to each reaction and mixed. Ten µL of neutralized assay sample were added and the plate was sealed with a silicon cap mat seal. The plate was first incubated at 96°C for 5 minutes and incubated at 50°C without agitation overnight in a conventional hybridization oven. A filter plate (Dura pore, Millipore part number MSBVN1250, 1.2 µm pore size) was prewetted with 75 µL 1× TMAC hybridization solution supplemented with 0.5% (w/v) BSA. The entire sample volume from the hybridization reaction was transferred to the filter plate. The hybridization plate was rinsed with 75 µL 1× TMAC hybridization solution containing 0.5% BSA and any remaining material was transferred to the filter plate. Samples were filtered under slow vacuum, with 150 µL buffer requiring about 8 seconds to evacuate. The filter plate was washed once with 75 µL 1× TMAC hybridization solution containing 0.5% BSA and the microspheres in the filter plate were resuspended in 75 µL 1× TMAC hybridization solution containing 0.5% BSA. The filter plate was protected from light and incubated on an Eppendorf Thermalmixer R for 5 minutes at 1000 rpm. The filter plate was then washed once with 75 µL 1× TMAC hybridization solution containing 0.5% BSA. 75 µL of 10 µg/mL streptavidin phycoerythrin (SAPE-100, MOSS, Inc.) in 1× TMAC hybridization solution was added to each reaction and incubated on Eppendorf Thermalmixer R at 25°C at 1000 rpm for 60 minutes. The filter plate was washed twice with 75 µL 1× TMAC hybridization solution containing 0.5% BSA and the microspheres in the filter plate were resuspended in 75 µL 1× TMAC hybridization solution containing 0.5% BSA. The filter plate was then incubated protected from light on an Eppendorf Thermalmixer R for 5 minutes, 1000 rpm. The filter plate was then washed once with 75 µL 1× TMAC hybridization solution containing 0.5% BSA. Microspheres were resuspended in 75 µL 1× TMAC hybridization solution supplemented with 0.5% BSA, and analyzed on a Luminex 100 instrument running XPonent 3.0 software. At least 100 microspheres were counted per bead type, under high PMT calibration and a doublet discriminator setting of 7500 to 18000. Primary Luminex readout data is available as a Supplementary File (Hybridization Data).

### QPCR read-out

Standard curves for qPCR were prepared in water ranging from 10^8^ to 10^2^ copies with 10-fold dilutions and a no-template control. Neutralized assay samples were diluted 40-fold into diH_2_O. The qPCR master mix was prepared at 2× final concentration (2× KOD buffer, 400 µM dNTP mix, 400 nM forward and reverse primer mix, 2× SYBR Green I and 0.5 U KOD EX). Ten µL of 2× qPCR master mix was added to 10 µL of diluted assay sample. qPCR was run on a BioRad MyIQ iCycler with 2 minutes at 96°C followed by 40 cycles of 96°C for 5 seconds and 72°C for 30 seconds.

### Pull-down assay

Pull-down assays were performed as described previously^3^.

## Supporting Information

Figure S1 **Demonstration of SOMAmer specificity by pull-down assay.** SOMAmers were incubated with target proteins, plasma, or target proteins spiked into plasma for 45 minutes. Protein/SOMAmer complexes were captured on magnetic streptavidin beads (MyOne C1), washed, and then treated with a mixture of NHS-biotin and NHS-AlexaFluor 647. Protein/SOMAmer complexes were photocleaved from beads and a portion fractionated on SDS gels (first set of 3 lanes, marked “equilibrium”). Protein/SOMAmer complexes were then adsorbed to monomeric avidin agarose beads, washed, and then eluted with 2 mM biotin in SB17. Complexes were captured a third time onto magnetic streptavidin beads (MyOne C1) substituted with a bound biotinylated-primer complementary to the 3′ fixed region of the SOMAmer. Not all SOMAmer complexes can be captured onto these beads since the 3′ fixed regions of SOMAmers are sometimes inaccessible for annealing while bound to the target protein (as evident in the gels for MMP-7 and MMP-9). The complexes were eluted by increasing the pH to 12, and then neutralized. Portions were fractionated on SDS gels (second set of 3 lanes). Shown are purified target protein spiked into buffer (lanes 1), purified target protein spiked into 10% plasma (lanes 2), and 10% plasma with no spike (lanes 3).
(TIF)

Figure S2 **Precision profiles and limits of quantification of plate- and bead-based assays.** Eight individual measurements of fluorescent signal as a function of analyte concentration in buffer were made for each of nine analytes in multiplexed format. For the dose-response curves (left of each panel), the average RFU at each concentration is denoted by the blue markers and the eight individual measurements used to compute each average are denoted by the red markers plotted on the four parameter curve fit (solid blue line). Precision profiles (right of each panel) were computed with two different methods: (1) by calculating the variance in computed concentrations (blue, bottom left of each panel) and (2) by calculating the variance in log RFU (assay response, top right of each panel) combined with the slope of the standard curve (red). Left-hand panels were generated in plate-based (SOMAPanel) format. Right-hand panels were generated in bead-based (SOMAscan) format. Limits of quantification were defined as points on the curve in which the coefficient of variation (CV) exceeded 20%.
(TIF)

Figure S3 **QPCR readout of plate-based assay eluates.** Portions of samples generated in the experiment for Table 2 were diluted and assayed by qPCR per Materials and Methods.
(TIF)

Figure S4 **Serum titration.** Serum was added at 0.078%, 0.156%, 0.313%, 0.625%, 1.25%, 2.50%, 5.00%, and 10.0% to the 9-plex SOMAmer panel. The plate-based assay was performed in semi-automated format and analyte signal measured as described in Materials and Methods.
(TIF)

Figure S5 **Minimum multiplex capacity of plate-based assay platforms.** Sixty-one SOMAmers recognizing analytes identified as biomarkers on the bead-based SOMAScan platform were combined. Serum was added at 0.011%, 0.035%, 0.11%, 0.35%, 1.1%, 3.45%, 10.9%, and 34.5% v/v, and analyte signal measured as described in Materials and Methods. The log of the ratio of analyte signal at 10.9% and 0.011% was calculated, and plotted as a cumulative distribution function. Analyte signals at 10.9% serum are elevated at least 2.8-fold over those at 0.011% serum for all sixty-one biomarkers.
(TIF)

Figure S6 **Hybridization data.** Raw data used to generate Figures 2, 3, 4 and 5 is provided in spreadsheet format.
(XLSX)
