# Supplementary figures and images for: From SOMAmer-Based Biomarker Discovery to Diagnostic and Clinical Applications: A SOMAmer-Based, Streamlined Multiplex Proteomic Assay
Source: PLoS One. 2011 Oct 17;6(10):e26332. doi: 10.1371/journal.pone.0026332 (PMC3195687; doi:10.1371/journal.pone.0026332)

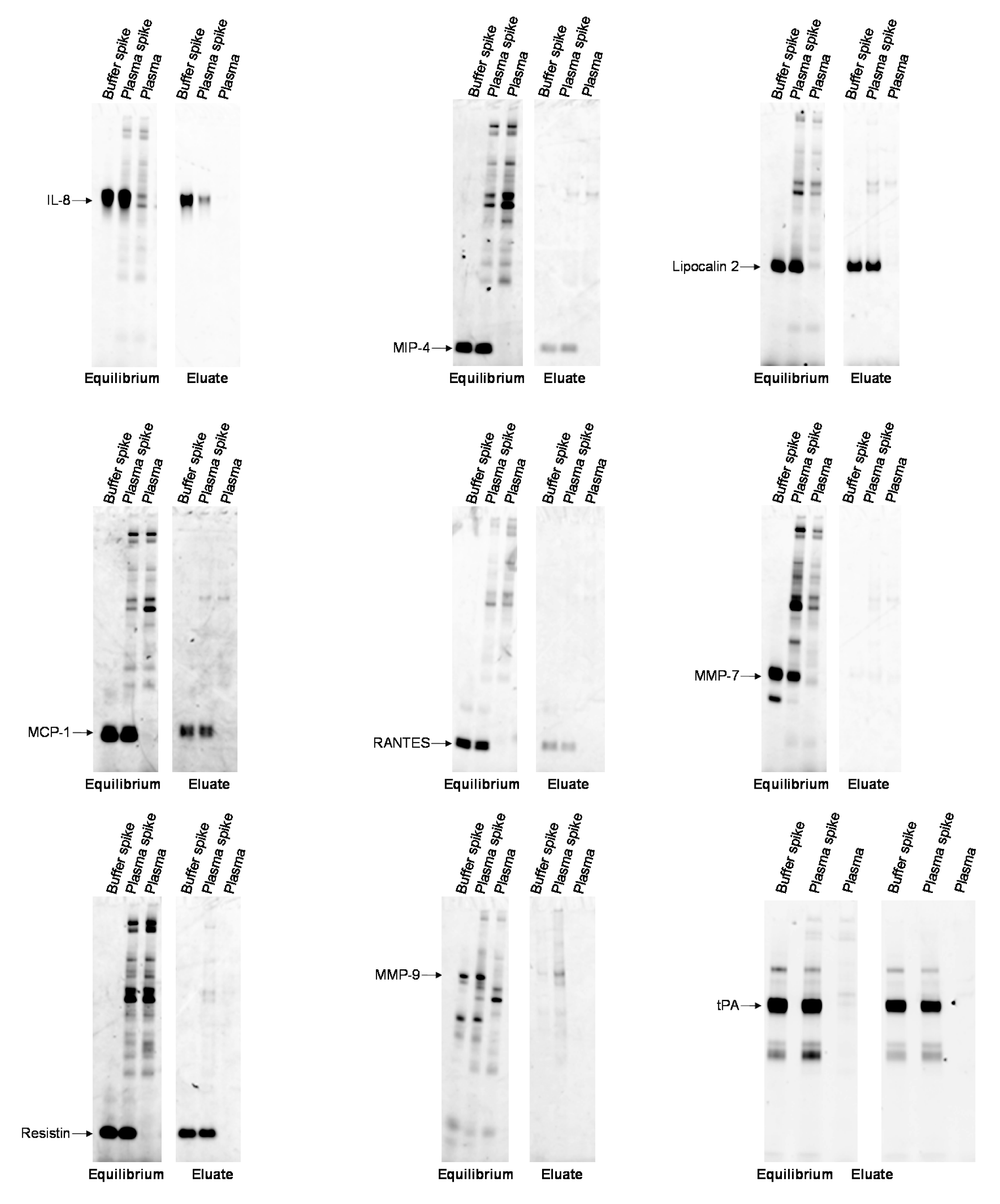

Supplement: Figure S1 — Demonstration of SOMAmer specificity by pull-down assay. SOMAmers were incubated with target proteins, plasma, or target proteins spiked into plasma for 45 minutes. Protein/SOMAmer complexes were captured on magnetic streptavidin beads (MyOne C1), washed, and then treated with a mixture of NHS-biotin and NHS-AlexaFluor 647. Protein/SOMAmer complexes were photocleaved from beads and a portion fractionated on SDS gels (first set of 3 lanes, marked “equilibrium”). Protein/SOMAmer complexes were then adsorbed to monomeric avidin agarose beads, washed, and then eluted with 2 mM biotin in SB17. Complexes were captured a third time onto magnetic streptavidin beads (MyOne C1) substituted with a bound biotinylated-primer complementary to the 3′ fixed region of the SOMAmer. Not all SOMAmer complexes can be captured onto these beads since the 3′ fixed regions of SOMAmers are sometimes inaccessible for annealing while bound to the target protein (as evident in the gels for MMP-7 and MMP-9). The complexes were eluted by increasing the pH to 12, and then neutralized. Portions were fractionated on SDS gels (second set of 3 lanes). Shown are purified target protein spiked into buffer (lanes 1), purified target protein spiked into 10% plasma (lanes 2), and 10% plasma with no spike (lanes 3). (TIF) [file pone.0026332.s001.tif]

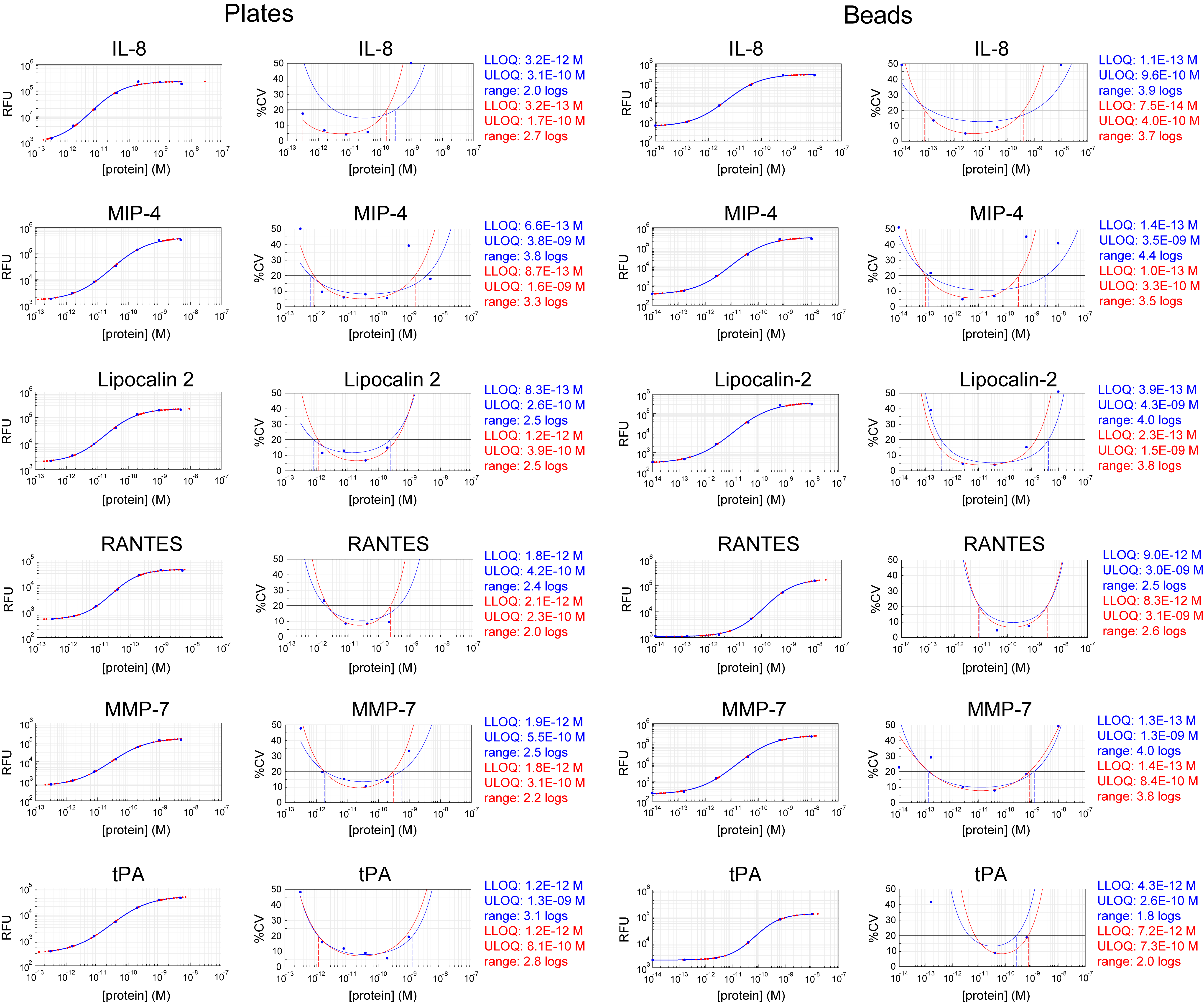

Supplement: Figure S2 — Precision profiles and limits of quantification of plate- and bead-based assays. Eight individual measurements of fluorescent signal as a function of analyte concentration in buffer were made for each of nine analytes in multiplexed format. For the dose-response curves (left of each panel), the average RFU at each concentration is denoted by the blue markers and the eight individual measurements used to compute each average are denoted by the red markers plotted on the four parameter curve fit (solid blue line). Precision profiles (right of each panel) were computed with two different methods: (1) by calculating the variance in computed concentrations (blue, bottom left of each panel) and (2) by calculating the variance in log RFU (assay response, top right of each panel) combined with the slope of the standard curve (red). Left-hand panels were generated in plate-based (SOMAPanel) format. Right-hand panels were generated in bead-based (SOMAscan) format. Limits of quantification were defined as points on the curve in which the coefficient of variation (CV) exceeded 20%. (TIF) [file pone.0026332.s002.tif]

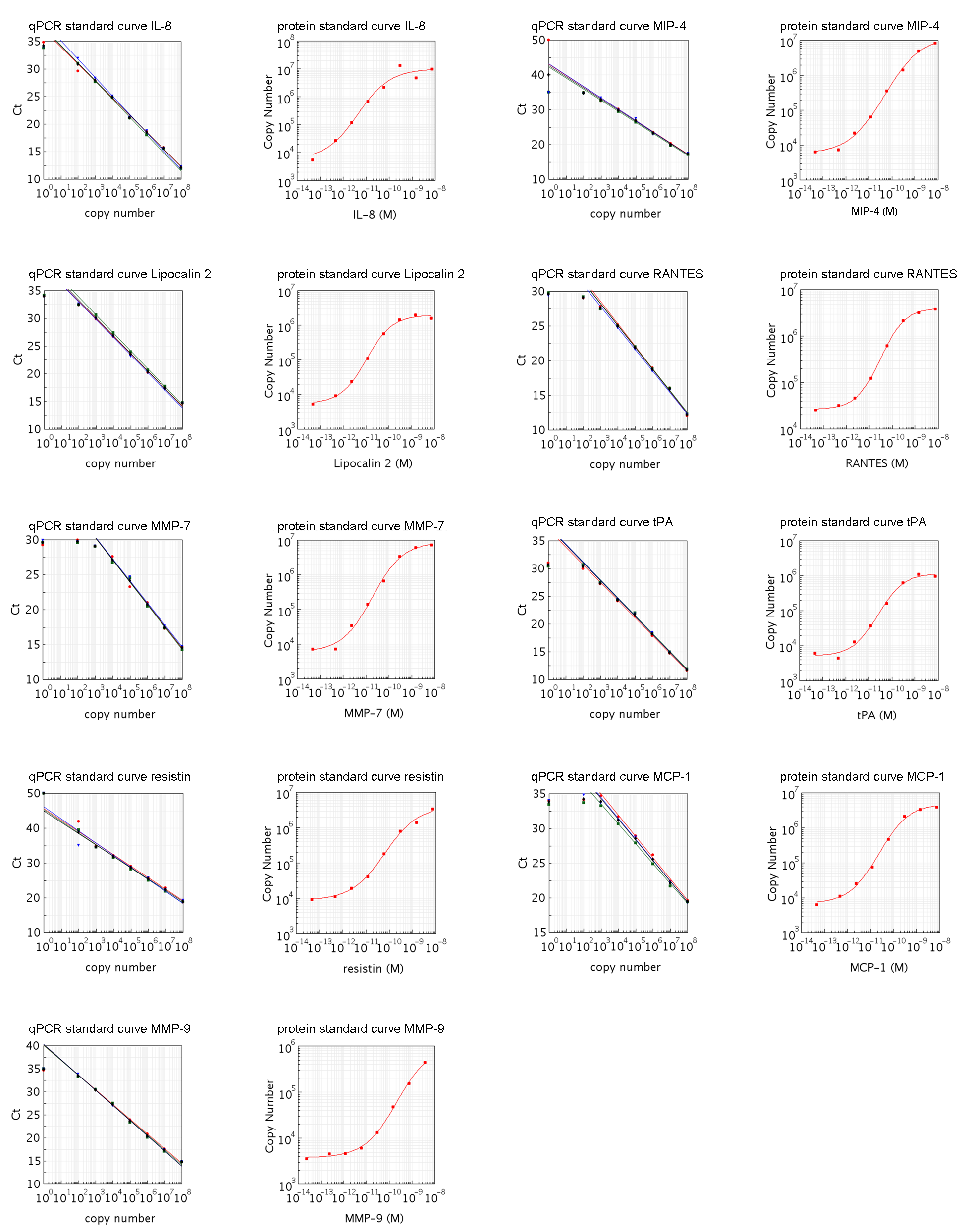

Supplement: Figure S3 — QPCR readout of plate-based assay eluates. Portions of samples generated in the experiment for Table 2 were diluted and assayed by qPCR per Materials and Methods. (TIF) [file pone.0026332.s003.tif]

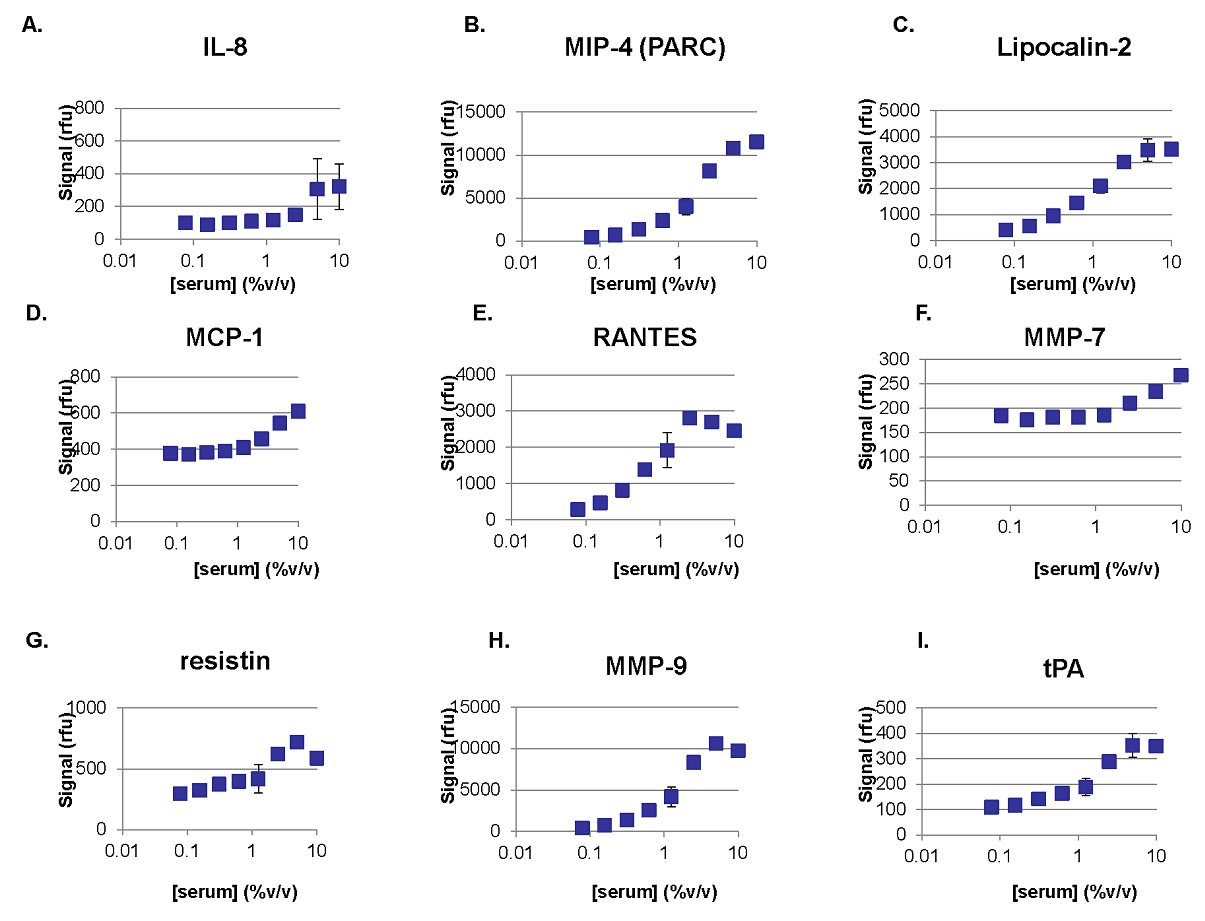

Supplement: Figure S4 — Serum titration. Serum was added at 0.078%, 0.156%, 0.313%, 0.625%, 1.25%, 2.50%, 5.00%, and 10.0% to the 9-plex SOMAmer panel. The plate-based assay was performed in semi-automated format and analyte signal measured as described in Materials and Methods. (TIF) [file pone.0026332.s004.tif]

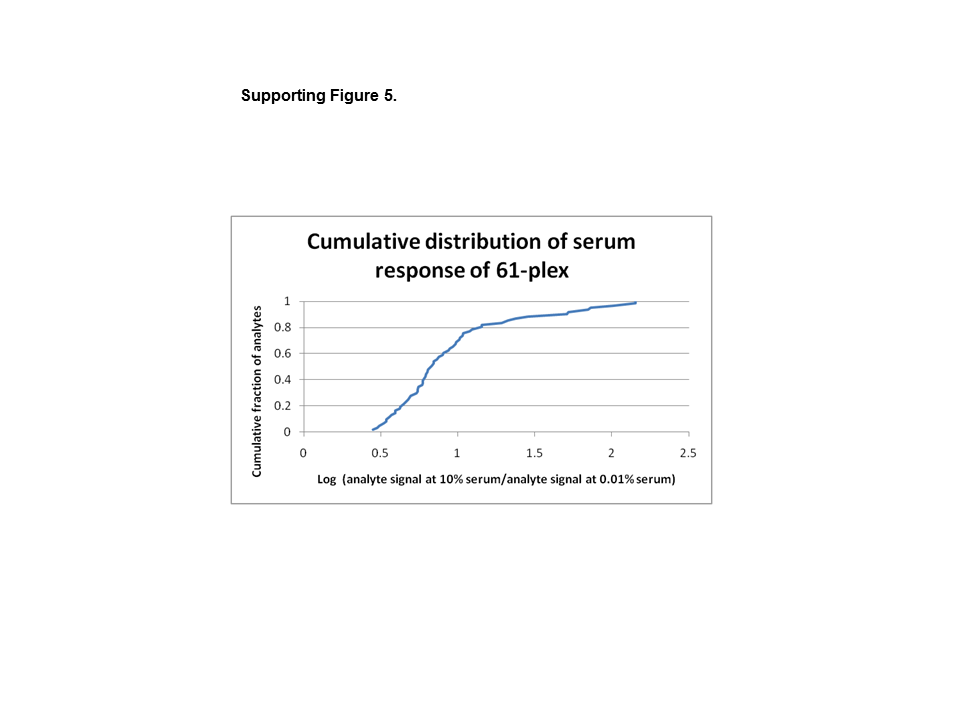

Supplement: Figure S5 — Minimum multiplex capacity of plate-based assay platforms. Sixty-one SOMAmers recognizing analytes identified as biomarkers on the bead-based SOMAScan platform were combined. Serum was added at 0.011%, 0.035%, 0.11%, 0.35%, 1.1%, 3.45%, 10.9%, and 34.5% v/v, and analyte signal measured as described in Materials and Methods. The log of the ratio of analyte signal at 10.9% and 0.011% was calculated, and plotted as a cumulative distribution function. Analyte signals at 10.9% serum are elevated at least 2.8-fold over those at 0.011% serum for all sixty-one biomarkers. (TIF) [file pone.0026332.s005.tif]
